# Validity and reliability of short forms of parental-caregiver perception and family impact scale in a Telugu speaking population of India

**DOI:** 10.1186/s12955-016-0433-7

**Published:** 2016-03-01

**Authors:** Santhosh Kumar, Jeroen Kroon, Ratilal Lalloo, Newell W. Johnson

**Affiliations:** Population and Social Health Research Programme, Menzies Health Institute Queensland and School of Dentistry and Oral Health, Griffith University, Queensland, Australia; School of Dentistry, The University of Queensland, Queensland, Australia; School of Dentistry and Oral Health, Griffith University, Queensland, Australia; Population and Social Health Research Programme, Menzies Health Institute Queensland, Griffith University, Queensland, Australia

**Keywords:** Children, Cross-cultural adaptation, Oral health related quality of life, Parent-caregiver perception questionnaire, Telugu, Translation

## Abstract

**Background:**

Parental-Caregiver Perception Questionnaire (P-CPQ) and Family Impact Scale (FIS) are commonly used measures to evaluate the parent’s perception of the impact of children’s oral health on quality of life and family respectively. Recently, shorter forms of P-CPQ and FIS have been developed. No study has sought to validate these short forms in other languages and cultures. This study aimed to evaluate the validity and reliability of FIS, 8 and 16-item P-CPQ in a Telugu speaking population of India.

**Methods:**

For this cross-sectional study, a multi-stage random sampling technique was used to recruit 11–13 year-old schoolchildren of Medak district, Telangana, India and their parents (*n* = 1342). Parents were approached with questionnaires through their children who underwent clinical examinations for dental caries, fluorosis and malocclusion. The translated versions underwent pilot testing (*n* = 40), test-retest reliability was also assessed (*n* = 161).

**Results:**

The overall summary scale and subscales of the short forms of P-CPQ and FIS failed to discriminate between the categories of dental caries severity. Also, malocclusion status was not related to the domain or overall scores of both the short forms of P-CPQ. There were significant differences in subscale and overall scores of 16 and 8-item P-CPQ and FIS between the fluorosis categories_._ Both 16 and 8-item P-CPQ summary scales were significantly related to parent’s global rating of oral health (16-item, *r* = 0.30, *p* < 0.01; 8-item, *r* = 0.28, *p* < 0.01) and overall wellbeing (16-item, *r* = 0.22, *p* < 0.01; 8-item, *r* = 0.22, *p* < 0.01), thereby exhibiting good construct validity. However, the correlation of emotional and social wellbeing scales of short forms of P-CPQ and FIS with global ratings was of low strength. Cronbach’s alphas for FIS, 16-items and 8-items P-CPQ scales were 0.78, 0.83 and 0.71 respectively, while the Intra-Class Correlation coefficients were 0.752, 0.812 and 0.816 respectively. Cronbach’s alphas for most of the subscales of short forms of P-CPQ were less than 0.7.

**Conclusions:**

The overall scales of 16 and 8-items P-CPQ scales demonstrated good construct validity while the construct validity of FIS was questionable. Discriminant validity of all the three instruments was good only in relation to fluorosis. Overall scales of all three short forms exhibited acceptable internal consistency and reliability on repeated administrations.

## Background

Child Oral Health Quality of Life (COHQOL) is a battery of instruments, proposed to comprise self-administered, age-specific Oral Health Related Quality of Life (OHRQoL) questionnaires [1]. These are the Child Perception Questionnaire (CPQ) for children in the age groups 5–7 years, 8–10 years [2] and 11–14 years [1], the Parental-Caregiver Perception Questionnaire [3] (P-CPQ) and the Family Impact Scale [4] (FIS). Both the P-CPQ and FIS are administered to parents. P-CPQ evaluates the parent’s or caregiver’s perception of the impact of their child’s oral health on his/her quality of life [5] while FIS assesses the impact of child’s oral condition on the family [4].

Literature suggests that children below 6 years of age are not capable of abstract thinking and of reasoning the timing of past events. Therefore it is recommended to use adults as proxy to report the impact of dental disease in children [6]. However, P-CPQ has been developed for administering to parents of children even older than 6 years as a supplement to children’s self-report of oral health and wellbeing (CPQ) [3]. The main rationale for proposing the supplemental use of P-CPQ with CPQ is that parents or caregivers are intimately involved in the health care of their children [3]. Child health comprises not only physical growth but also emotional development and social functioning, both within and outside the family. It is thus plausible to consider Quality of Life (QoL) from the perspective of both parents and their children [7]. Parents and caregivers are the major influence on a child’s health behaviours [8]. Seeking treatment for children is also driven by parents’ perceptions of a child’s illness or suboptimal QoL [3, 8–10].

Oral diseases can have negative impact on functional, social and psychological wellbeing of both children and their families [11]. This emphasises why family impact needs to be recorded along with self-reported and parental perception of QoL in children. FIS assists to assess caregiver-burden bias when using parental reports of child’s health as these can be influenced by the emotional and physical burden caused to parents by their child’s condition [4].

The 31 item P-CPQ, which consists of four domains or subscales (oral symptoms, functional limitations, emotional wellbeing and social wellbeing) and 14 item FIS were developed a decade ago and have been found to be valid in an English speaking Canadian population [3, 4]. P-CPQ has also been successfully translated and adapted in Brazil [8, 12], Peru [13], China [14] and Germany [15]. The psychometric properties of P-CPQ were also found to be acceptable in a UK population [16]. FIS has also been tested for cross-cultural acceptability in Brazil [17, 18] and China [14].

Recently a short form of FIS (8 items) and two forms of P-CPQ (16 items with four domains of four items each; 8 items with four domains of two items each) have been developed with acceptable validity and reliability in a New Zealand population [19]. No study has sought to validate these short forms in other languages and cultures or analysed the psychometric properties of either the short or long versions of P-CPQ and FIS in the Indian subcontinent, specifically in Telugu language. Telugu is estimated to be spoken by 74 million people across two southern states (Telangana and Andhra Pradesh) of India. It is the most spoken language after Hindi and Tamil in India [20].

Shorter versions were preferred as they had good psychometric properties [19] similar to the original longer versions [3, 4] and cause less burden to the respondents [19], specifically in the context of our study where the parents were required to answer a battery of questionnaires.

This study aimed to evaluate the validity and reliability of FIS and of the 8 and 16 item P-CPQ in a Telugu speaking population of India.

## Methods

### Sampling and study population

This study was conducted as part of PhD project of SK. Ethical approval was granted by the Griffith University Human Research Ethics Committee in Australia and the ethics committee of Panineeya Institute of Dental Sciences & Research Centre, Hyderabad, India. Subjects for this study included parents and school children of Medak district in the state of Telangana, India. Medak is one of the 23 districts of Telugu speaking states, Telangana and Andhra Pradesh, India. Children were recruited by a multi stage random sampling procedure. At first stage, 9 subdistricts (administrative divisions) were randomly selected from a total of 46 sub-districts in Medak. At second stage, schools proportional to the total number of schools in each subdistrict were randomly selected (36 schools were selected from a total of 455 schools in the district). Later, all 6^th^ grade children (age 11 to 14) from the selected schools were invited to participate. All the invited children expressed interest and were provided with consent forms to be signed by the parents and also child and parent versions of the questionnaires. Consenting children underwent clinical examinations by a single examiner (SK) in the schools for dental caries, fluorosis and malocclusion. Dental caries was recorded in the permanent dentition and deciduous dentition using the Decayed Missing and Filled Teeth (DMFT) and dft indices respectively [21] and fluorosis using Dean’s Fluorosis index [22]. World Health Organization criteria was adopted for caries diagnosis [23]. In addition, malocclusion status was recorded: a subject was considered to have malocclusion when he/she had any kind of malocclusion classified as Class I/II/III by Angle [24], or gross orthodontic problems which requires orthodontic treatment. The child questionnaire consisted of items about health-related behaviours, Telugu translation of Child Perception Questionnaire for 11- to 14-year-old children (CPQ_11–14_) and a child’s perception of his/her relationship with parents. The Parent questionnaire consisted of items related to socio-demographics, family environment, health-related behaviours, P-CPQ, FIS, parent’s relationship with their children (Parent Child Relationship Questionnaire [25]) and parenting style (Parental Authority Questionnaire-Revised [26]). Children were requested to preferably have the questionnaire completed by the mother. If this was not feasible it could be completed by the father or other caregiver. To encourage participation a free oral hygiene kit was provided to each child and an incentive of a mobile recharge card worth 50 Indian Rupees was given to parents. After 2 weeks children in four randomly chosen schools were approached to evaluate the reliability of the previously completed questionnaires. Children were again provided with questionnaires to be completed by their parents and were instructed to return these within 3 days. For this study, P-CPQ and FIS from parent questionnaires and clinical examination data were only used.

### Adaptation of short forms of Telugu P-CPQ and FIS

In order to achieve culturally acceptable and conceptually equivalent P-CPQ and FIS, specific guidelines [27] for cross cultural adaptation of self-report measures were followed. English versions of the short forms of P-CPQ and FIS were translated into Telugu by two independent translators. The principal author was one of the translators. A single translated version was developed with consensus from both translators which was then back translated into English by two independent translators. An expert committee consisting of all the translators, a public health dentist and two school teachers was then formed to develop the final version of short forms of Telugu P-CPQ and FIS. The responses for the Telugu short forms of P-CPQ and FIS were similar to that of the English versions which ranged from “never =0” to “every day or almost every day = 4” on a 5 point Likert scale. In addition, two statements on global ratings of oral health and overall wellbeing were added to the questionnaire to evaluate the general perception of parents or caregivers of their child’s oral health and its effect on overall wellbeing. The responses for these two statements ranged from “excellent = 0” to “poor = 4” and “not at all = 0” to “very much = 4” respectively on a five point Likert scale. The translated version was administered to 40 parents for assessing the content validity of the translated questionnaires.

### Statistical analysis

Statistical analysis was conducted using IBM SPSS Statistics for Windows, Version 22.0. (New York, IBM Corp). For discriminant validity, subscale and overall scores of P-CPQ and FIS were compared between the categories of oral disease levels. As the data was skewed, non-parametric statistics were used. Mann Whitney *U* test was used to compare the scores across the categories of fluorosis and malocclusion while Kruskal Wallis H test was used for evaluating statistical differences between the categories of caries severity. Construct validity of the translated questionnaires were tested by assessing the correlations of subscale and overall scores with the global ratings of oral health and overall wellbeing. Spearman Correlation coefficient was used for this purpose. In addition, adjusted correlation using partial ‘r’ was calculated after adjusting for potential confounders which included gender of the parent or caregiver, Soico-economic status (SES), fluorosis, malocclusion and dental caries status. Correlations below 0.20 were considered weak, 0.20–0.30 as medium and >0.30 as high [28]. Internal consistency of subscale and overall scales was assessed using Cronbach’s alpha (values of 0.7 and more are considered acceptable [29]) and test retest reliability was evaluated using Intra class correlation co-efficient (ICC). ICC of 0.61–0.8 was considered good and >0.8 was considered excellent [30].

## Results

A total of 1580 questionnaires were distributed, of which 1342 questionnaires were returned with complete data. All the children who returned the completed questionnaires underwent clinical examination (*n* = 1342). The age of the study population ranged from 11 to 14 years and more than half were boys (59 %). Approximately two thirds (64.6 %) of the questionnaires were completed by the mother followed by father (33 %) and other carer (2.4 %). Of 180 questionnaires that were re-distributed to parents after 2 weeks, 161 completed questionnaires were returned.

The overall summary scale and subscales of the short forms of P-CPQ and FIS failed to discriminate between the categories of dental caries severity (Table 1). Higher scores were reported for FIS by parents of children with malocclusion. However, malocclusion status was not related to the domain or overall scores of the 16 item P-CPQ. There were striking differences in parent reported subscale scores and summary scores of the 16 item P-CPQ and FIS between the fluorosis categories. Parents of children with moderate and severe fluorosis reported higher impact of oral health on children’s quality of life and family.

**Table 1:** Discriminant validity of the 16 item P-CPQ and FIS in relation to dental caries, malocclusion and fluorosis

	Oral symptoms	Functional limitations	Emotional well being	Social well being	16 item P-CPQ	FIS
	Median (IQR)	Median (IQR)	Median (IQR)	Median (IQR)	Median (IQR)	Median (IQR)
Dental caries (DMFT + dft)						
DMFT+ dft = 0	3(5)	2(4)	1(4)	0(2)	7(11)	2(7)
DMFT+ dft =1–3	3(5)	2(5)	1(5)	0(3)	9(14)	3(7)
DMFT+ dft >3	3(5)	2(3)	1(5)	0(2)	7(11)	3(9)
Malocclusion						
No	3(5)	2(4)	1(4)	0(2)	7(13)	2(6)^*^
Yes	3(5)	2(4)	1(4)	0(2)	7(12)	3(8)
Fluorosis						
None to mild	3(5)^*^	2(4)^*^	1(3)^*^	0(2)^*^	7(11)^*^	2(7)^*^
Moderate to severe	6(5)	4(5)	4(8)	1(5)	15.5(18)	5(9)

^*^Mann Whitney U test, *p* = 0.021

^*^Mann Whitney *U* test, Wilcoxon W, *p* < 0.001

No differences in subscale and overall scores of the 8 item P-CPQ were found between the dental caries severity categories (Table 2). Similar to the 16 item P-CPQ, the 8 item P-CPQ and its domains failed to discriminate between the malocclusion categories, but significant differences were found between the fluorosis categories.

**Table 2:** Discriminant validity of the 8 item P-CPQ in relation to dental caries, malocclusion and fluorosis

	Oral symptoms	Functional limitations	Emotional well being	Social well being	8 item P-CPQ
	Median (IQR)	Median (IQR)	Median (IQR)	Median (IQR)	Median (IQR)
Dental caries					
DMFT + dft =0	2(3)	1(3)	0(2)	0(0)	4(7)
DMFT + dft =1–3	2(3)	1(3)	0(2)	0(1)	5(8)
DMFT + dft >3	2(3)	0(3)	0(3)	0(0)	4(6)
Malocclusion					
No	2(3)	1(3)	0(2)	0(1)	4(6)
Yes	2(3)	1(3)	0(2)	0(1)	4(8)
Fluorosis					
None to mild	2(3)^*^	1(2)^*^	0(2)^*^	0(0)^*^	4(6)^*^
Moderate to severe	3(2)	2(3)	1.5(4)	0(3)	8(10)

^*^Mann Whitney *U* test, *p* < 0.001

Table 3 demonstrates that 16 and 8 item P-CPQ summary scales were significantly positively related to parent’s global rating of children’s oral health (16 item P-CPQ, *r* = 0.30, *p* < 0.01; 8 item P-CPQ, *r* = 0.28, *p* < 0.01) and overall wellbeing (16 item P-CPQ, *r* = 0.22, *p* < 0.01; 8 item P-CPQ, *r* = 0.22, *p* < 0.01). These correlations remained significant even after controlling for confounding variables. Although significant, FIS had poor correlation with overall oral health (*r* = 0.11, *p* < 0.01) and wellbeing (*r* = 0.11, *p* < 0.01). The correlations of emotional and social wellbeing subscales of both 16 and 8 items P-CPQ with the global ratings were also of low strength.

**Table 3:** Correlation of the 16 and 8 item P-CPQ overall and subscale scores with global self-rating of oral health and overall wellbeing

	Global rating of oral health		Global rating of overall wellbeing	
	*r*	Partial *r*	*r*	Partial *r*
16 item P-CPQ				
Oral symptoms	0.333^a^	0.330^a^	0.215^a^	0.206^a^
Functional limitations	0.247^a^	0.249^a^	0.159^a^	0.145^a^
Emotional well being	0.192^a^	0.198^a^	0.158^a^	0.150^a^
Social well being	0.136^a^	0.117^a^	0.114^a^	0.111^a^
Total P-CPQ	0.302^a^	0.303^a^	0.220^a^	0.205^a^
8 item P-CPQ				
Oral symptoms	0.275^a^	0.280^a^	0.211^a^	0.198^a^
Functional limitations	0.251^a^	0.251^a^	0.166^a^	0.132^a^
Emotional well being	0.176^a^	0.173^a^	0.135^a^	0.130^a^
Social well being	0.076^a^	0.064^a^	0.103^a^	0.097^a^
Total P-CPQ	0.279^a^	0.289^a^	0.221^a^	0.202^a^
FIS	0.111^a^	0.105^a^	0.112^a^	0.115^a^

*r* – Spearman correlation coefficient

Partial *r* – correlation coefficient adjusted for gender of the parent or caregiver, SES, fluorosis, malocclusion and dental caries

^a^Correlation is significant at the 0.01 level (2-tailed)

Table 4 presents the reliability statistics of the 16 and 8 item P-CPQ along with FIS. Cronbach’s alphas for overall scale of the 16 item P-CPQ was 0.839. Oral symptoms (0.629) and functional limitations (0.611) subscales had lower values and also social well-being (0.626). Cronbach’s alphas were very low for the 8 item P-CPQ ranging from 0.299 (Social wellbeing) to 0.710 (Total P-CPQ). FIS had an acceptable internal consistency value of 0.782. ICC’s for test-retest reliability for all the domains and overall scales were all acceptable with the lowest ICC observed for oral symptoms domain (0.735) of the 16 items P-CPQ and the highest ICC for the social wellbeing domain (0.902) of the 8 item P-CPQ.

**Table 4:** Internal consistency, test-retest reliability and descriptive data of overall OHRQoL and its subscales

	Number of items	Mean ± SD	Range	ICC	95 % CI	Cronbach’s alpha
16 item P-CPQ						
Oral symptoms	4	3.73 ± 3.25	0–16	0.735	0.655–0.799	0.629
Functional limitations	4	2.57 ± 2.93	0–16	0.818	0.759–0.863	0.611
Emotional well being	4	2.44 ± 3.15	0–15	0.776	0.706–0.831	0.735
Social well being	4	1.36 ± 2.28	0–13	0.895	0.859–0.922	0.626
Total P-CPQ	16	10.10 ± 9.04	0–53	0.812	0.751–0.858	0.839
8 item P-CPQ						
Oral symptoms	2	1.93 ± 1.83	0–8	0.759	0.685–0.818	0.425
Functional limitations	2	1.54 ± 1.87	0–8	0.779	0.710–0.833	0.387
Emotional well being	2	1.21 ± 1.80	0–8	0.748	0.672–0.809	0.645
Social well being	2	0.59 ± 1.20	0–7	0.902	0.869–0.927	0.299
Total P-CPQ	8	5.30 ± 4.85	0–26	0.816	0.757–0.862	0.710
FIS	8	4.44 ± 5.08	0–24	0.752	0.676–0.812	0.782

## Discussion

In this study we evaluated the psychometric properties of FIS along with the 8 and 16 item P-CPQ. P-CPQ and FIS were found to have acceptable reliability and validity by the developers. However, when using QoL instruments in different languages and cultures along with the translation, questionnaire are supposed to be adapted culturally to maintain the content validity at a conceptual level [27]. Therefore, guidelines prescribed by Beaton et al., [27] for translation and cross-cultural adaptation were strictly followed in this study. For instance the item “had a hard time paying attention in school” was slightly corrected to “had difficulty in paying attention in school” and “acted shy or embarrassed” to “felt shy or embarrassed in front of others” for semantic equivalence. In addition, in the statement “avoided smiling or laughing when around other children”, the word smiling was deleted as both smiling and laughing have approximately the same meaning in the dialect of this study population.

In order to evaluate the content validity of the instruments in this setting, questionnaires were administered to a sample of 40 parents. Personal interviews were conducted with each parent after completing the questionnaire to find if they had any problems or suggestions for improvement. Subsequent to this, changes were made to the statement on missed school to “missed school because of pain, appointments with the doctor or dentist or for taking dental treatment” as majority of the parents got confused whether the question was related to general health or specifically oral health.

Dental caries severity failed to influence the subscale or overall scores of any of the parental reports in this study. This might be because, clinical indicators measure disease while the concept of OHRQoL intends to evaluate overall health and well-being [17]. Further, Locker and Slade described that the weak association of subjective ratings with clinical variables might be due to the type or nature of the disease, confounding effect of socio-demographic variables and difference in perceptions levels between the subjects [31]. Dental caries in particular, when in early stages of the disease, might not affect the child’s ability to perform his/her day to day activities [17]. Therefore, for dental caries to have an impact on quality of life, it most likely requires pulpal involvement, which was a rare finding in this child population.

No differences were observed in P-CPQ domain and overall scores between the malocclusion categories, similar to findings from a previous study [16] in a UK population, however some differences were observed in few domains between Chinese children attending paediatric and orthodontic clinics [14]. Parents of children with moderate and severe forms of fluorosis reported higher impacts in all the subscales of P-CPQ and also FIS. The reason for contrast in relationship of parental perceptions with malocclusion and fluorosis might be because of the awareness of the parents about self-correcting malocclusion in children of this age with mixed dentition in contrast to the permanent nature of fluorosis.

Global ratings of oral health and overall wellbeing were significantly positively correlated to FIS, the 8 and 16 item P-CPQ and their subscales in the expected direction. However, the strength of association observed between FIS and the global ratings was weak which makes the construct validity of Telugu FIS questionable. Further, the emotional and social well-being subscales of both the 16 items and 8 items P-CPQ weakly correlated with global oral ratings. Weaker correlation of these subscales with overall global ratings is evident from a previous study [16] conducted in the UK. In general, overall scales of 16 items and 8 items P-CPQ demonstrated good construct validity, the 16 items P-CPQ had the highest correlation followed by the 8 item P-CPQ and FIS. Contrasting findings have been reported from previous validation studies with the majority finding an association with global ratings [14, 16, 17] while only one study from Brazil reported no correlation [7].

Although the internal consistency scores of FIS and the 16 and 8 item P-CPQ overall scales were above 0.7, which is acceptable, the values observed in this study (0.78, 0.83 and 0.71 respectively) are less than those observed by developers of the short forms where Cronbach’s alpha values of 0.85, 0.89 and 0.82 for FIS and the 16 and 8 item P-CPQ respectively were reported [19]. Oral symptoms had less than acceptable reliability estimate which is in accordance with previous studies on cross-cultural adaptation of P-CPQ [14, 16] and that was observed by the developers of P-CPQ [3]. Also, the Cronbach’s alphas for functional limitations and social wellbeing was less than 0.70 as observed by Marshman et al., [16]. All the domains of the short form of 8 item P-CPQ had unacceptable Cronbach’s alpha values which might be due to less number of items in each subscale, evidence suggests that reliability estimates increases with the scale length [32]. The internal consistency values for subscales have not been reported by the developers of short forms to enable comparison. However ICC’s for test-retest reliability for all the domains and overall scales were good to excellent in the range of 0.735 (oral symptoms domain of the 16 item P-CPQ) to 0.902 (social wellbeing domain of the 8 item P-CPQ).

In order to reduce the sampling variance, schools proportional to total number of schools in each sub district were selected. However, this study is not free of limitations. Firstly, study population were recruited from only one district. Studies on wider populations from few randomly chosen districts of Telugu speaking Indian states are recommended to further evaluate the psychometric properties of P-CPQ and FIS. Another limitation is that only private schools were considered for inclusion in this study. This is because, a greater proportion of parents of children in government schools are illiterate. According to data collected in 2014, more than half of the mothers and approximately one third of the fathers of children in public schools have never been to school [33]. Therefore, if the study also included government school children, the response rate would have been poor limiting the generalizability of the study findings. Further, the reading levels of children enrolled in government schools in India are “low” [34] and thus they might not be able to complete the child questionnaire. Lastly, Angle’s classification was used to assess the malocclusion; a comprehensive measure like Index of Treatment Needs would have been more relevant.

## Conclusions

The overall scales of 16 and 8-items P-CPQ scales demonstrated good construct validity while the correlation of FIS with global ratings was of low strength which makes its construct validity questionable. All the three scales failed to discriminate between the caries severity categories and the short forms of P-CPQ failed to differentiate the categories of malocclusion. Discriminant validity of all three scales and their subscales was good in relation to fluorosis. The overall scales of all three short forms (16 and 8 items P-CPQ and FIS) exhibited acceptable internal consistency and reliability on repeated administrations as assessed by Cronbach’s alpha and ICC respectively. However, the internal consistency reliability of most of the subscales of short forms of P-CPQ was below the acceptable level which requires further investigation.
